# Substance abuse treatment and psychiatric comorbidity: do benefits spill over? analysis of data from a prospective trial among cocaine-dependent homeless persons

**DOI:** 10.1186/1747-597X-1-27

**Published:** 2006-09-11

**Authors:** Stefan G Kertesz, Alok Madan, Dennis Wallace, Joseph E Schumacher, Jesse B Milby

**Affiliations:** 1 Division of Preventive Medicine, University of Alabama at Birmingham, School of Medicine and the Deep South Center on Effectiveness at the Birmingham Veterans' Affairs Medical Center; 1530 3rd Ave South MT 608 Birmingham, Alabama 35294, USA; 2 Department of Psychiatry, University of North Carolina School of Medicine, Medical School Wing E, CB#7205 Chapel Hill, North Carolina 27599-7205, USA; 3 Rho Federal Systems, Inc., Chapel Hill, North Carolina 27517, USA; 4 Division of Preventive Medicine, The University of Alabama at Birmingham, School of Medicine. 1530 3rd Ave South MT 616 Birmingham, Alabama 35294, USA; 5 Department of Psychology, The University of Alabama at Birmingham, Division of Preventive Medicine, The University of Alabama at Birmingham, 1530 3rd Ave South CH 415 Birmingham, Alabama 35294, USA

## Abstract

**Background:**

Comorbid psychiatric illness can undermine outcomes among homeless persons undergoing addiction treatment, and psychiatric specialty care is not always readily available. The prognosis for nonsubstance abuse psychiatric diagnoses among homeless persons receiving behaviorally-based addiction treatment, however, is little studied.

**Results:**

Data from an addiction treatment trial for 95 cocaine-dependent homeless persons (1996–1998) were used to profile psychiatric diagnoses at baseline and 6 months, including mood-related disorders (e.g. depression) and anxiety-related disorders (e.g. post-traumatic stress disorder). Treatment interventions, including systematic reinforcement for goal attainment, were behavioral in orientation. There was a 32% reduction in the prevalence of comorbid non-addiction psychiatric disorder from baseline to 6 months, with similar reductions in the prevalence of mood (-32%) and anxiety-related disorders (-20%) (p = 0.12).

**Conclusion:**

Among cocaine-dependent homeless persons with psychiatric comorbidity undergoing behavioral addiction treatment, a reduction in comorbid psychiatric disorder prevalence was observed over 6 months. Not all participants improved, suggesting that even evidence-based addiction treatment will prove insufficient for a meaningful proportion of the dually diagnosed homeless population.

## Background

In 2004, homeless persons in the United States accounted for 175,300 admissions to substance abuse treatment facilities receiving public funding [1], with recent cocaine use reported in 46% of those admissions [2]. Comorbid psychiatric disorders are an important complicating factor in the treatment of homeless drug users. In a national survey, 3/4 of homeless persons with a past-year drug problem also had a current nonsubstance-related mental health problem [3]. These figures are comparable to the general population of persons seeking treatment for drug use disorders. In the National Epidemiologic Survey on Alcohol and Related Conditions (2001–02), among persons who sought treatment for a current (past-year) drug use disorder, 60% had an independent mood-related disorder (MRD) and 43% had an independent anxiety-related disorder (ARD) during the same period [4].

Recent initiatives on homelessness make it important to understand what psychiatric outcomes can be obtained from behavioral addiction treatment programs serving dually diagnosed homeless persons. Initiatives of the United States federal government include a policy focus on ending chronic homelessness [5-9], and the instigation of 10-year homeless planning processes among 222 communities across the United States (Personal Communication, Mary Ellen Hombs, United States Interagency Council on Homelessness, August 3, 2006). Outcomes for homeless persons are also of interest because 80% of homeless single adults with nonaddiction mental health problems do not receive services for those problems [10]. Further, only 35% of substance abuse treatment facilities provide formal treatment for persons with comorbid mental health disorders [11], meaning that formally integrated dual diagnosis treatment programs, while potentially ideal [12], are likely to remain inaccessible for many dually diagnosed homeless persons.

Whether behavioral treatments for substance abuse in the homeless [13,14] confer benefit for comorbid mental illness remains unclear. Some reports describe reductions in psychiatric symptoms among persons undergoing addiction treatment, potentially due to resolution of post-drug dysphoric symptoms (including the post-cocaine "crash"[15]), or remission of substance-induced mental disorders [16]. Such data are not entirely informative for homeless policy, however, due to: (a) with exceptions[12], insufficient representation of homeless persons in reported samples; (b) a lack of formal diagnostic assessment at the treatment follow-up; and (c) a paucity of studies regarding formal behavioral addiction treatment approaches. Behavioral addiction treatments, defined as manualized nonpharmacologic interventions for substance abuse [17], include modalities such as community reinforcement, rewards for abstinence or other goal attainment, and cognitive behavioral therapy. Such approaches can be deployed in publicly funded addiction treatment programs that receive homeless persons. However, a review of the efficacy of behavioral therapies among dually diagnosed cocaine dependent persons noted that empiric data are notable mainly for their absence [18].

In response to a paucity of empirically validated treatment options for dually diagnosed (non-psychotic) homeless persons, we performed a secondary analysis of data from a randomized behavioral addiction treatment trial for homeless, non-psychotic, cocaine-abusing individuals, focusing on non-substance abuse psychiatric outcomes. The original trial intervention randomly assigned a behavioral contingency management approach (including abstinence contingent housing and paid vocational rehabilitation) to half of the participants while offering intensive behavioral day treatment (including psychosocial, cognitive behavioral, and rewards for nonsubstance-related goal attainment) to all participants. Substance use improved in both trial arms, but improved more in the contingency management group, as has been reported [19,20].

There was plausible basis to anticipate improvement in non-substance abuse psychiatric diagnoses. Vocational rehabilitation activities included social reinforcement, including vouchers for purchase of goods in response to goal accomplishment (for both trial arms). Individuals within the contingency-managed housing trial arm additionally had access to program-provided housing and work therapy dependent on the results of negative urine toxicology screens. The treatment program activities therefore had elements of behavioral activation, access to reinforcers, and distraction from negative maladaptive cognitions, features typical of cognitive-behavioral therapies [21,22]. Further, it bore resemblance to milieu therapy, which has a record of efficacy in depression [23].

The present examination sought to describe what improvement, if any, transpired for non-addiction psychiatric disorders after treatment was offered for 6 months. While this analysis takes account of trial arm, intensive day treatment was offered to all participants in this study, and this analysis assessed improvements in psychiatric status for persons with mood-related disorders and anxiety-related disorders for participants in both trial arms.

## Methods

The original treatment trial was conducted between April 1995 and August 1996 [19].

### Participants

Participants in the clinical trial were recruited from Birmingham Health Care (BHC), the largest health care agency for homeless persons in Birmingham, Alabama. Inclusion criteria were: 1) homeless according to McKinney Act criteria for homelessness [24]; 2) self-reported cocaine use in the past two weeks with a DSM-III-R diagnoses of cocaine abuse/dependence at baseline assessment; 3) endorsed significant symptoms of psychological distress based on a Symptoms Checklist 90-R scale t-score > 70[25]; 4) intended to stay in Birmingham for 12 months; 5) capable of providing informed consent; 6) no symptoms of severe medical or psychiatric disorder requiring immediate hospitalization; and 7) willing to participate in interventions and assessments.

One hundred sixty-three individuals were screened and found eligible to participate in the study. However, 16 did not attend baseline testing, 5 were found ineligible after reviewing screening assessments, and 1 refused participation. The remaining 141 individuals were randomly assigned to 1 of 2 treatment groups, as reviewed below. All participants provided informed consent, received modest compensation, and this study had the approval of the Institutional Review Board of the University of Alabama at Birmingham.

### Design and intervention

Participants were randomized to 1 of 2 interventions: Enhanced Day Treatment (DT+) and Day Treatment alone (DT). The trial intervention was a manualized, behavioral day treatment program occurring in 3 phases. During Phase I (months 0–2), all participants engaged in a substance abuse day treatment program from 7:30 am to 2:30 pm, including lunch and transportation to and from the treatment center. Individuals participated in group and individual therapy, and underwent urine drug testing. Day treatment emphasized individualized goal setting and review of goal attainment in major life domains (housing, employment, mental health, etc), with weekly vouchers provided for accomplished goals.

Upon achieving 2 weeks of abstinence, participants in the DT+ trial arm also received abstinent-contingent, rent-free, furnished apartments. However, a positive urine screen on random, twice-weekly testing resulted in immediate eviction of the individual from the rent-free apartment and transportation to a shelter. Individuals could return to program-provided housing once they re-established abstinence (2 consecutive negative urine toxicologies). Individuals in the DT arm did not have the option of abstinent-contingent housing but were free to pursue any other housing options available to them.

During Phase II (months 3–6), both DT+ and DT participants engaged in aftercare, consisting of weekly 1.5-hour group therapy sessions that emphasized goal setting and relapse prevention. Urine was screened at least once weekly. Individual counseling was available as needed. For individuals in the DT+ group, abstinent-contingent housing remained available for a rent of $162 per month. Rent could be earned through employment or program-provided abstinent-contingent work therapy. Individuals in the DT arm did not have the option of abstinent-contingent housing. While their specific lodging experiences were not recorded, many used local shelters and boarding and recovery homes.

### Primary outcome measures

The primary outcome measure for this analysis was the presence of current non-psychotic, non-substance abuse/dependence Axis I psychiatric diagnoses (e.g. depression, anxiety, etc.) at baseline and 6 months. Baseline diagnostic evaluations were completed 7–12 days following enrollment into the study. This delay was designed to reduce diagnostic imprecision resulting from post-cocaine dysphoric symptoms (including the post-cocaine "crash"), which are typically most intense during the first week after cessation [15,26].

Diagnostic assessments, lasting approximately 30 minutes to 1 hour, were conducted by a psychology post-doctoral fellow or faculty member, who assigned diagnoses based on a clinical diagnostic interview, structured with the help of a computer-administered DSM-III-R Checklist [27]. The checklist is designed to assist doctoral-level clinicians in establishing diagnoses for 22 Axis I disorders as well as one Axis II disorder, antisocial personality disorder. It served as a diagnostic clinical guide in the original Epidemiologic Catchment Area (ECA) Survey [28]. Its psychometric properties were established based on agreement of diagnoses with psychiatrist and lay administration of the Diagnostic Interview Schedule [29]. Specificities of all diagnoses were 90% or better, while sensitivities ranged from 47% to 100% [30]. The Checklist, based on DSM-III-R, did not explicitly provide a method to delineate independent from secondary comorbid psychiatric diagnoses (unlike instruments based on the DSM-IV [31,32]. However, consistent with the DSM-III-R, diagnostic evaluators were expected to delineate primary disorders based on a global, albeit unguided, clinical judgment. Clinical judgment was also applied to participants with symptoms outside of the scope of the instrument (e.g. trichotillomania).

Each participant provided data at baseline and at the end of Phase II (i.e. at the 6-month follow-up). For purposes of profiling the baseline distribution of diagnoses, individuals were categorized into 1 of 4 diagnostic groups. The first group consisted of individuals with only anxiety-related disorders (ARDs) (e.g. post-traumatic stress disorder, generalized anxiety disorder, and others). The second group consisted of individuals with only mood-related disorders (MRDs) (e.g. depression, dysthymia, and others). The third group consisted of individuals with both MRDs and ARDs (Combo). The remaining individuals had neither ARD nor MRD comorbid diagnoses (None). See Table 1 for details.

**Table 1:** Most Frequent Baseline Diagnoses in the Analytic Cohort (n = 95)^1^

***Disorder***	***Frequency***	***Percentage^2^***
Major Depressive Disorder	35	36.8%
Post-traumatic Stress Disorder	21	22.1%
Dysthymia	12	12.6%
Generalized Anxiety Disorder	11	11.6%
Simple Phobia	10	10.5%
Social Phobia	9	9.5%
Adjustment Disorder	7	7.4%
Bipolar Disorder	7	7.4%
Obsessive-Compulsive Disorder	4	4.2%
None	26	27.4%

Notes for Table 1

1. The analytic cohort is restricted to those participants available at baseline and follow-up at 6 months (N = 95 of 127 original participants).

2. Percentages add up to greater than 100% because participants were eligible for multiple diagnoses. Each of the following diagnoses were applied only once: Trichotillomania, Agoraphobia without Panic Disorder, Alcohol-induced Psychotic Disorder, Anxiety Disorder Not Otherwise Specified (NOS), Delusional Disorder NOS, Dementia Alzheimer's Type, Dementia NOS, Dissociative Disorder NOS, Eating Disorder NOS, Insomnia NOS, Pain Disorder, Paraphilia NOS, and Tic Disorder NOS.

### Covariates

In modeling changes in the prevalence of the 2 psychiatric disorder categories (ARDs and MRDs) over 6-months, analyses took into account several measures that could plausibly be related to these outcomes and might be construed as confounders or explanatory variables. Age, education, gender and race were included as demographic covariates. Abstinence was defined as the longest number of consecutive weeks abstinent, as measured by drug-negative urine toxicologies, during the time frame encompassed by the present analysis (i.e. 6 months). Trial arm assignment (DT and DT+) and treatment attendance were also included as covariates to ascertain the impact of treatment intensity on outcome. Attendance was categorized, based on review of the biphasic distribution of participation in outpatient day treatment in Phase I (months 1–2) and Phase II (3–6), thusly: "low" (low attendance in both Phase I and Phase II), "medium" (high attendance in Phase I or Phase II, but not both Phases) and "high" (high attendance in both Phase I and Phase II).

### Data analysis

To assess the relationship between disorder type (ARD and MRD) and time (0 months, 6 months), this analysis included data for individuals available at baseline and 6 months. To assess the potential for selection bias, baseline characteristics for included participants to those participants unavailable at six months were compared with Mann-Whitney U tests and chi-squared tests as indicated.

To evaluate whether mood and anxiety disorder outcomes differed for participants who contributed observations at baseline and follow-up, the outcome of psychiatric disorder (presence/absence) was modeled in relation to disorder type (ARD and MRD) and time (0 months and 6 months) using an extension of the generalized linear model, a generalized estimating equation [33]. To allow model specification that could address the questions of interest, 4 binary outcomes were generated for each participant based on disorder type at each time period. For many readers, the inclusion of the *type *of disorder and the *time *of the observation interval as predictors for a generic outcome of "psychiatric disorder present/absent" will not seem intuitive, but this approach permits statistically testing whether psychiatric disorder presence was dependent on observation interval (i.e., baseline versus 6 months later) and whether it differed for anxiety-related as opposed to mood-related disorders. This approach is analogous to an extension of the McNemar test often used for paired binary outcomes (the ARD and MRD diagnoses) in a longitudinal setting. This GEE approach incorporates the between-diagnostic-category and temporal correlation inherent to this study design.

A hierarchical modeling approach [34] was employed to analyze the data. A base model (including only the observation interval and type of disorder) was specified, followed by 3 additional exploratory models, which varied in regard to the inclusion of 3 groups of plausible covariates chosen *a priori *(i.e. demographic, abstinence-related, and treatment intensity-related covariates). All 4 models included the disorder type and observation interval as predictors, co-represented as 4 predictor terms (ARD at baseline, ARD at 6 months, MRD at baseline, MRD at 6 months), along with a separate term for observation interval (baseline versus 6 months) representing the effect of interval time, independent of disorder type. Model 1 included only these 4 disorder/interval terms and the term for interval. Model 2 included these 5 terms, and the demographic covariates of gender, race, education and age. Model 3 included the same 5 terms as Model 1, and consecutive weeks of abstinence. Model 4 included the same 5 terms, two treatment intensity covariates [trial arm assignment (DT or DT+) and day treatment attendance]. Potential evidence of treatment benefit was sought through post hoc testing for the 3-way interactions of disorder type by time by treatment arm, and of disorder type by time by attendance. Recognizing that such analyses should be considered exploratory given the small numbers of subjects, p-values are offered as indicators of the likelihood of potential associations, rather than formal hypothesis testing.

SAS Version 8.0 (SAS Institute, 2001, Cary, NC) was the statistical software package used for all statistical analyses.

## Results

Of the initial 141 individuals enrolled in the study, 127 completed DSM-III-R assessments at baseline (DT+ = 69, DT = 58), and 95 of these 127 (74.8%) individuals provided complete data at the 6-month follow-up. Table 1 lists the prevalence of the most common disorders among trial participants at baseline. A comparison of analyzed participants (persons providing complete data at baseline and at the 6-month follow-up, n = 95) to individuals providing only baseline data (n = 32), revealed no significant differences between groups on demographic variables, diagnostic categorization, and baseline abstinence (Table 2). The final sample consisted predominantly of young/middle-aged (mean age = 37.7 ± 7.1 years), African-American (86.3%) males (74.7%) with some college education (mean education = 13.0 ± 2.0 years). All participants met criteria for 1 or more current substance abuse/dependence disorders, with cocaine (96.9%) and alcohol disorders (57.8%) occurring most frequently, as has been detailed in another report [35].

**Table 2:** Comparison of Individuals Providing Only Baseline Data and Individuals Providing Complete Data at Baseline and at Six Months' Follow-Up

Characteristic^1^	*Baseline Only *(n = 32)	*Available at Baseline and 6 months (n = 95)*	*Statistical Test^2^*	*p*
Age	38.97 (7.96)	37.65 (7.10)	U = 1391.5	*0.59*
Race (% AA)	71.9	86.3	χ^2 ^= 3.49	*0.06*
Gender (% male)	68.8	74.7	χ^2 ^= 0.44	*0.51*
Education	12.93 (3.45)	13.03 (2.02)	U = 1465	*0.76*
Diagnostic Category at Baseline (%)			χ^2 ^(df 3) = 0.08	0.99
ARD Only (%)	9.4	10.5		
MRD Only (%)	28.1	26.3		
Combo (%)	31.3	32.6		
None (%)	31.3	30.5		
Abstinent (%)^3^	100.0	98.9	χ^2 ^= 0.34	*0.56*
Anti-social Personality Disorder (%)	28.1	31.6	χ^2 ^= 0.13	*0.71*

Table Notes

1. Abbreviations: ARD = anxiety-related disorder; MRD = mood-related disorder; Combo = presence of both ARD and MRD; AA = African American.

2. Continuous variables are compared with the Mann-Whitney U-Test, and categorical variables are compared with the χ^2 ^test (df = 1 for all comparisons except where noted).

3. Abstinent (%) refers to abstinence at baseline, by urine test in the first few days after entering treatment

At baseline, 69 of 95 individuals (73%) also met criteria for at least 1 current additional non-substance abuse/dependence, non-psychotic, Axis I psychiatric disorder. As shown in Table 3, 26% of participants had MRD with no ARD, 11% had ARD but no MRD, 33% had both disorder types, while 3% had a disorder not falling into either category. Despite efforts to exclude individuals at the time of screening with psychotic disorders, two individuals enrolled in the study later met criteria for alcohol-induced psychotic disorder and delusional disorder NOS, respectively. The most common MRDs were Major Depressive Disorder (n = 35), Dysthymia (n = 12), Adjustment Disorder (n = 7) and Bipolar Disorder (n = 7). The most common ARDs were Post-traumatic Stress Disorder (n = 21), Generalized Anxiety Disorder (n = 11), and Simple Phobia (n = 10).

**Table 3:** Frequency of Disorders, By Category of Mood-versus Anxiety-Related Disorder at Baseline and 6 Months

***Category***	***Baseline [n (%)]***	***Follow-up [n (%)]***
*Mood-Related Disorder (MRD)*	25 (26%)	15 (16%)
MRD Only	24	14
MRD + Other^1^	1	1
*Anxiety-Related Disorder (ARD)*	10 (11%)	12 (13%)
ARD Only	10	12
ARD + Other^1^	0	0
*Combination (MRD and ARD)*	31 (33%)	10 (11%)
MRD + ARD	26	9
MRD + ARD + Other^1^	5	1
*Neither MRD nor ARD*	29 (31%)	58 (61%)
Other Only^1^	3	2
*Total with Any Comorbid Disorder*	**69 (73%)**	**39 (41%)**
*Total with No Comorbid Disorder*	**26 (27%)**	**56 (59%)**
**Total Participants**	**95 (100%)**	**95 (100%)**

Notes

1. "Other" refers to disorders other than Mood- or Anxiety-Related Disorders and includes Trichotillomania, Alcohol-induced Psychotic Disorder, Delusional Disorder NOS, Dementia Alzheimer's Type, Dementia NOS, Dissociative Disorder NOS, Eating Disorder NOS, Insomnia NOS, Pain Disorder, Paraphilia NOS, and Tic Disorder NOS.

**Figure 1:**
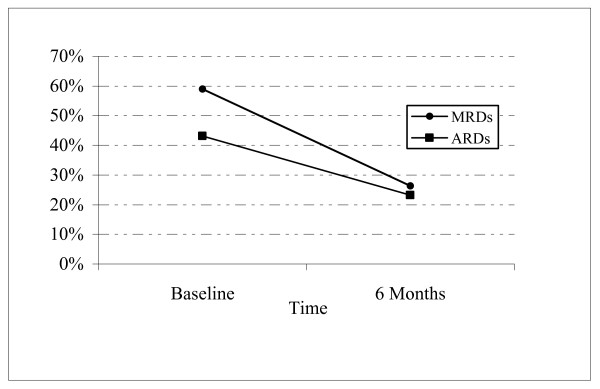
**Percentage of Participants with Mood-Related Disorders (MRDs) and Anxiety-Related Disorders (ARDs) at Baseline and at 6-month Follow-up**. Ninety-five homeless, cocaine-dependent treatment trial participants were diagnostically assessed at baseline and at 6 months' follow-up for the presence of Mood-Related Disorders (MRDs) or Anxiety-Related Disorders (ARDs), using a clinical interview conducted by a trained psychologist. MRDs included disorders such as depression and bipolar disorder. ARDs included disorders such as post-traumatic stress disorder and generalized anxiety disorder (for details, see Methods). The figure depicts the prevalence of each disorder type (MRDs and ARDs) among this sample at baseline and 6 months. The 32% absolute decline in prevalence of non-addiction psychiatric disorders from baseline to 6 months corresponds to -26% change in a statistical model adjusting for disorder type, and accounting for multiple observations per person. (χ^2 ^[df 1] = 30.49, p < 0.001). A test of whether the decline in MRD prevalence from 0 to 6 months differed from the decline for ARDs was nonsignificant, (χ^2 ^[df 1] = 2.48, p = 0.12).

At 6 months, 39 participants (41%) had at least 1 non-substance abuse/dependence, non-psychotic, Axis I psychiatric disorder, with similar proportions having MRDs and ARDs (16% with MRD but no ARD; 13% with ARD but no MRD; 11% with both MRD and ARD; 2% with a disorder in neither category). Figure 1 shows the proportion of participants diagnosed with MRDs and ARDs  at baseline and at 6-month follow-up. Table 3 displays the frequency for  each disorder type separately and in combination.

The GEE analyses (Model 1) revealed a significant independent effect of observation interval, consistent with 26% lower prevalence of non-substance abuse psychiatric disorders at 6 month follow-up compared to baseline, independent of the type of disorder (χ^2 ^[df 1] = 30.49, p < 0.001). The model-derived baseline-to-6-month change in the proportion of participants with MRDs (-33%), when tested in Model 1, was not statistically significantly greater than the change for ARDs (-20%) (χ^2 ^[df 1] = 2.48, p = 0.12). The reduction in prevalence of MRDs and ARDs, from 0 to 6 months, is depicted in Figure 1. The addition of demographic variables to the model (Model 2) revealed that female gender was associated with a 19% greater likelihood of having any disorder (χ^2 ^[df 1] = 2.32, p = 0.007). No other demographic variables were associated with psychiatric disorder presence. The abstinence indicator variables (Model 3) were not associated with psychiatric disorder presence (all p > 0.50, data not shown). Models including treatment intensity covariates (trial arm and attendance) did not suggest clear associations with either variable (Model 4). There was no significant 3-way interaction of disorder type by time by treatment assignment (χ^2 ^[df 3] = 0.25, p = 0.97) or disorder type by time and by attendance (χ^2 ^[df 6] = 3.87 p = 0.69).

## Discussion

Psychiatric comorbidity is common among homeless individuals presenting for addiction treatment. Data regarding the prognosis for comorbid psychiatric disorders among cocaine abusers undergoing behaviorally-based addiction treatment are infrequent [17,18], especially among homeless samples. In this study at baseline, 73% of a homeless cocaine-abusing sample qualified for 1 or more comorbid disorders. Common diagnoses included depression and post-traumatic stress disorder. This proportion fell to 41% at 6 months. Multivariable analyses did not demonstrate associations of trial arm or treatment intensity (including attendance) with improvement for either disorder type, but these statistical models were quite substantially underpowered, relying upon 3-way interaction terms with small subject numbers.

More than one interpretation for these findings is entirely possible. For instance, these findings may reflect a "spillover psychiatric benefit" for a behavioral addiction treatment program that offered elements of behavioral activation, social and tangible rewards, and milieu therapy. Conversely, evidence of improvement over time may simply indicate that (a) emotional dysphoria characterizes many persons early in the course of addiction treatment, and (b) dysphoric symptoms early in treatment can suggest psychiatric diagnoses, and (c) such symptoms tend to abate. Only a non-treatment control arm, something not present in this study and not frequently available in research involving homeless populations, could definitively disentangle these and other possible explanations.

Our findings may be compared to studies that have shown improvements in psychiatric symptoms among dually diagnosed inpatients [12,36] and community-dwelling outpatients [37] who received integrated treatment programs with services for comorbid addiction and nonaddiction psychiatric problems. However those studies relied on brief measures of psychiatric symptoms and functioning [38], rather than the more specific diagnostic assessments used in this study, and they involved programs that formally integrated addiction and psychiatric services, a potentially desirable approach that is not consistently funded in many communities. Since this trial was conducted in a state where neither Medicaid nor free psychiatric care are freely accessible to single adults with nonpsychotic mental illness, these findings may reflect what results can be expected from behavioral addiction treatment, absent formal psychiatric treatment services.

An interpretive challenge for this study is the degree to which the findings reflect the remission of substance-induced psychiatric disorders or withdrawal symptoms, rather than remission of independent psychiatric disorders. The diagnostic method used here makes the first interpretation somewhat less plausible. Evaluations were formally structured, conducted by doctoral-level individuals, and occurred 7–12 days after last use (e.g. after the period when the most prominent mood dsyphoria is typically reported by cocaine users in treatment [26]). Nonetheless there remains debate regarding the applicability of psychiatric diagnostic terms to individuals early in addiction recovery [39], and future research in this area should include instruments that formally address the distinction of independent and secondary psychiatric disorders, such as the Psychiatric Research Interview for Substance and Mental Disorders (PRISM) [39,31].

Limitations to the present analysis, notably sample size and power, have been noted. In the homeless population, however, there is a lack of other studies that have accrued and subjected to formal psychological diagnosis a similarly-sized sample of dually diagnosed cocaine abusers up to 6 months after behavioral treatment.

Important strengths to this study include its being among the few to focus on psychiatric outcomes in dually diagnosed homeless individuals with non-psychotic disorders. Homeless individuals in particular represent a distinctly challenging group for the conduct of prospective research. For a study of homeless persons with crack-cocaine addictions and high rates of comorbidity, a 6-month follow-up rate of 74.8% compares favorably with similar studies [40,41].

## Implications

These findings should be relevant to policymakers, given ongoing governmental interest in both chronic homelessness and addiction treatment, coupled with limitations on access to specialty psychiatric care. A recent managed care consensus panel's recommendations for treating dually diagnosed individuals suggests that addiction treatment programs must be ready to respond to comorbid psychiatric disorders [42,43]. Problematically, few dual diagnosis treatment options are empirically validated among non-psychotic, homeless populations. Moreover, only 35% of addiction treatment programs offer programs and services for persons with comorbid mental illness [11]. The reduction in psychiatric disorder prevalence observed in this sample, if confirmed in future trials, suggests a potential secondary benefit to behavioral addiction treatment for some dually diagnosed homeless. However, over 40% of this cohort continued to qualify for nonaddiction psychiatric diagnoses at 6 months. Accordingly, even if evidence-based, comprehensive behavioral addiction treatment were to become the norm in publicly funded programs, many homeless individuals are still likely to require psychiatric specialty care in conjunction with addiction treatment.

## Contributions

SGK conceived of this analysis, interpreted findings and drafted the manuscript and redrafted in response to reviews.

AM conceived of this analysis, collected measures, interpreted findings and drafted the manuscript.

DW helped plan and conduct the analysis and revised the manuscript.

JES designed the original trial, conducted it, helped interpret findings and revised the manuscript.

JBM designed the original trial, conducted it, helped interpret findings and revised the manuscript.

All authors helped to revise and approved the final draft of the manuscript.

## Competing interests

The author(s) declare that they have no competing interests.
